# Supplementary figures and images for: A simulation-based evaluation of methods for estimating census population size of terrestrial game species from genetically-identified parent-offspring pairs
Source: PeerJ. 2023 Apr 12;11:e15151. doi: 10.7717/peerj.15151 (PMC10105560; doi:10.7717/peerj.15151)

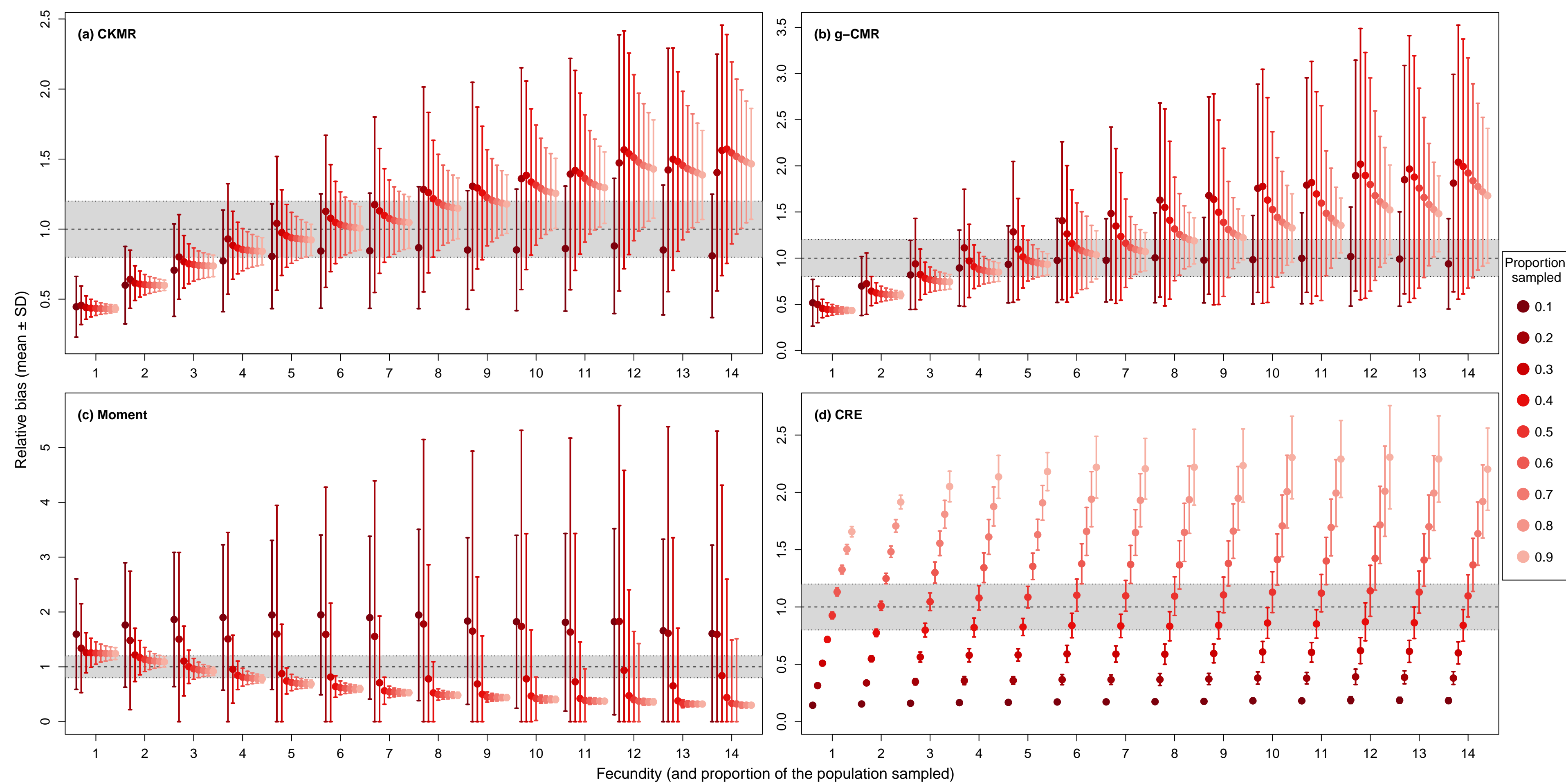

Supplement: Supplemental Information 3 — CKMR and CRE methods estimate adult population size while g-CMR and Moment methods estimate the whole population size and the breeding female population size, respectively. The long-dashed horizontal line represents the optimal value for an unbiased estimator, estimators above this line are overestimating the true population size while estimators below are underestimating it. Points in the grey area represent estimations within 20% of the true population size. [file peerj-11-15151-s003.pdf]

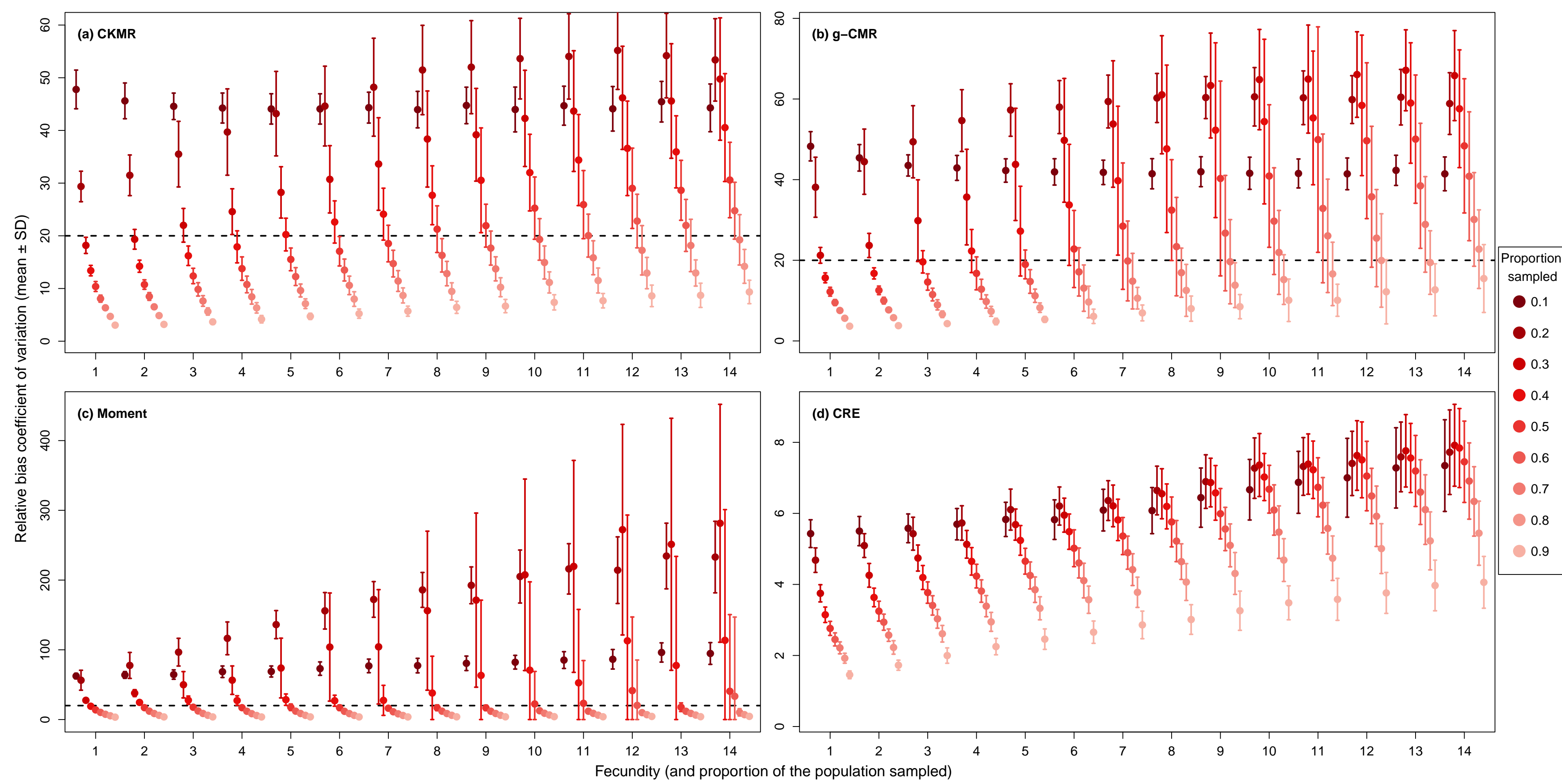

Supplement: Supplemental Information 4 — CKMR and CRE methods estimate adult population size while g-CMR and Moment methods estimate the whole population size and the breeding female population size, respectively. The long-dashed horizontal line represents the optimal value of 20% for a precise estimator, estimators above this line are too variable to be useful for wildlife management and conservation. [file peerj-11-15151-s004.pdf]

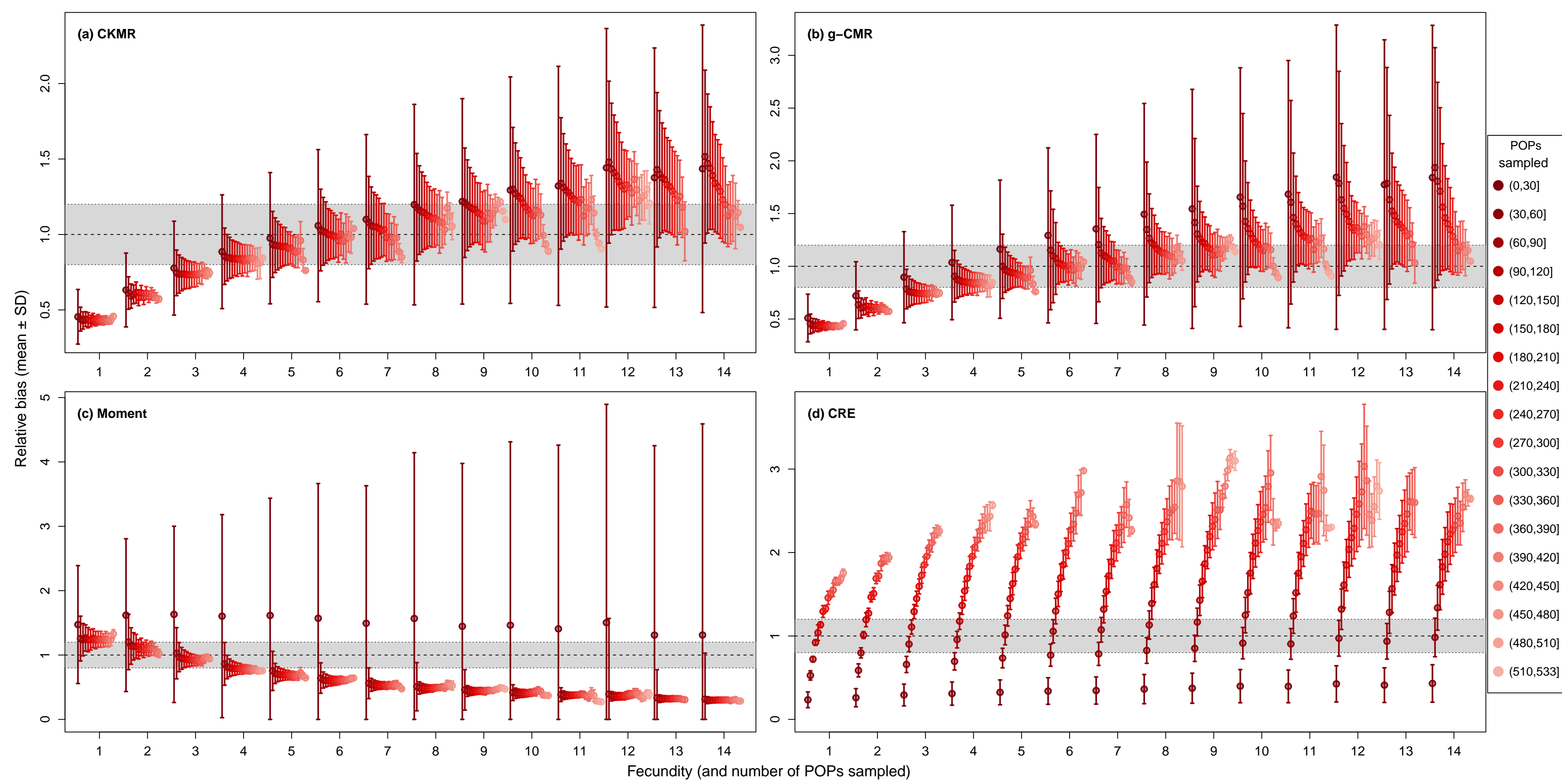

Supplement: Supplemental Information 5 — CKMR and CRE methods estimate adult population size while g-CMR and Moment methods estimate the whole population size and the breeding female population size, respectively. The long-dashed horizontal line represents the optimal value for an unbiased estimator, estimators above this line are overestimating the true population size while estimators below are underestimating it. Points in the grey area represent estimations within 20% of the true population size. [file peerj-11-15151-s005.pdf]
