# Supplementary material for: A simulation-based evaluation of methods for estimating census population size of terrestrial game species from genetically-identified parent-offspring pairs
Source: PeerJ. 2023 Apr 12;11:e15151. doi: 10.7717/peerj.15151 (PMC10105560; doi:10.7717/peerj.15151)
Supplement: Supplemental Information 6 [file peerj-11-15151-s006.docx]

**Supplemental information for**

**A simulation-based evaluation of methods for estimating census population size of terrestrial game species from genetically-identified parent-offspring pairs**

Jeremy Larroque, Niko Balkenhol

**S3 Close-Kin Mark-Recapture adjustment example**

In the naïve formula for the Close-Kin Mark-Recapture method (*i.e.*, second formula in Box 1b), the probability *P* that a randomly chosen adult-offspring pair is a parent-offspring pair solely depends on the size of the adult (*i.e.*, breeding) population. In other words, the chance that an individual in the sample is the offspring of an adult in the sample is *P* = 2/*N_A_*. Solving for adult population size, we can see that *N_A_* = 2/*P*. This probability is a basic assumption for the estimation of adult population sizes from close-kin data. However, for most applications, we need a more realistic estimation of this probability, for example because different ages or sexes have different reproductive outputs, or different probabilities of being sampled (*e.g.*, different probabilities of being harvested by hunters). Table S1 illustrates how correcting for *P* can improve population size estimates using the CKMR method.

**Table S1** Cartoon example to illustrate the importance of accurately estimating *P*, based on a population consisting of 15 adults and including 14 parent-offspring pairs. In the first row, all individuals are sampled, leading to a correct estimation of *N_A_* = 8. This estimation is also correct for row two, where fewer samples are taken, but the probability that an adult-juvenile pair is a parent-offspring pair is the same in the sample (*P_s_* = 0.25) and in the population (*P_p_* = 0.25). Thus, the assumption that *P* = 2/*N_A_* holds true so that population estimation is unbiased. In contrast, in rows three and four, *Ps* is lower than *Pp*, leading to biased estimates of *N_A_* = 9 and 12, respectively. We can correct for this bias by dividing *P_p_* by *P_s_* and multiplying the population estimates with the resulting correction factor. This leads to accurate *N_A_* estimates in all cases. In empirical studies, we do not know the true *P_p_*, hence we cannot apply such simple corrections. Instead, we have to estimate *P* as accurately as possible using biological information on fecundities, survival and sampling probabilities for different sexes and ages.

When age-specific fecundities for the study species are available, one way to derive more realistic values of *P* is by dividing, for each adult-offspring pair, the reproductive output of the adult *i* in the birth year of offspring *j* by the total reproductive output of adults in that year:

*P_(i is offspring of j)_* = $\frac{reproductuve output of adult j in year-of-birth of offspring i}{total reproductive output of adults in year-of-birth of offspring i}$

These estimations are usually done for both sexes separately, or for females only (Bravington et al. 2016).

Similarly, it is possible to include age- or sex-specific survival rates in the estimation of population sizes (*e.g.*, Ruzzante et al. 2019). The logic behind this is that any sample of *n* individuals will underestimate true population size in the year-of-birth of those individuals, simply because neither all individuals born in that year, nor their potential parents, may have survived long enough to be included in the sample. Thus, for more accurate estimations of population sizes, *P* again needs to be adjusted for the varying survival probabilities of offspring and their parents.

Several studies illustrate ways to derive estimations of *P* and population size that are statistically more robust and biologically more meaningful than the naïve estimators we used here, and all of these studies are based on the CKMR approach (*e.g.*, Hillary et al. 2016; Waples and Feutry 2022). This is not surprising, because CKMR methods are the most widely used among the four methods we tested. However, incorporating more realistic fecundities, survival and sampling probabilities should be possible for any method based on mark-recapture principles, including not only CKMR, but also the Moment estimator and g-CMR. While it is challenging to obtain reliable, year-specific age estimations and population-specific data on reproduction and survival for many terrestrial game species, we nevertheless believe that future studies should strive to improve the naïve estimators by accounting for more realistic demographic information for the study species. Doing so will further increase the potential of the tested estimators for application in terrestrial game species.

Bravington MV, Grewe PM, Davies CR (2016) Absolute abundance of southern bluefin tuna estimated by close-kin mark-recapture. Nature Communications 7:13162

Hillary RM, Bravington MV, Patterson TA, Grewe P, Bradford R, Feutry P, Gunasekera R, Peddemors V, Werry J, Francis MP, Duffy CAJ, Bruce BD (2018) Genetic relatedness reveals total population size of white sharks in eastern Australia and New Zealand. Scientific Reports 8:2661

Ruzzante DE, McCracken GR, Førland B, MacMillan J, Notte D, Buhariwalla C, Mills Flemming J, Skaug H (2019) Validation of close-kin mark–recapture (CKMR) methods for estimating population abundance. Methods in Ecology and Evolution 10:1445-1453


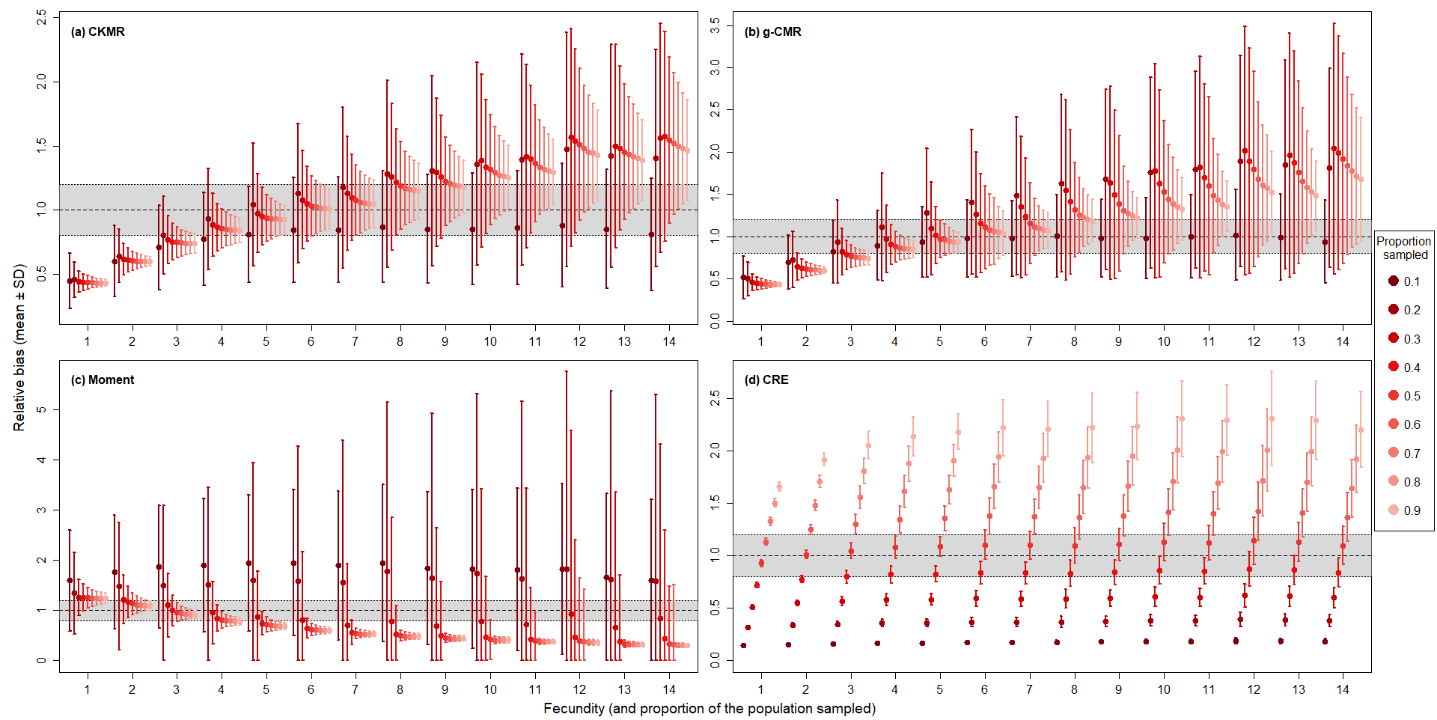


**Fig. S1** Relative bias (*N̂*/*N*, mean ± SD) as a function of the fecundity (ranging from 1 to 14), and the proportion of the population sampled ranging from (0.1 to 0.9) for the (a) CKMR, (b) g-CMR, (c) Moment and (d) CRE methods. CKMR and CRE methods estimate adult population size while g-CMR and Moment methods estimate the whole population size and the breeding female population size, respectively. The long-dashed horizontal line represents the optimal value for an unbiased estimator, estimators above this line are overestimating the true population size while estimators below are underestimating it. Points in the grey area represent estimations within 20% of the true population size.


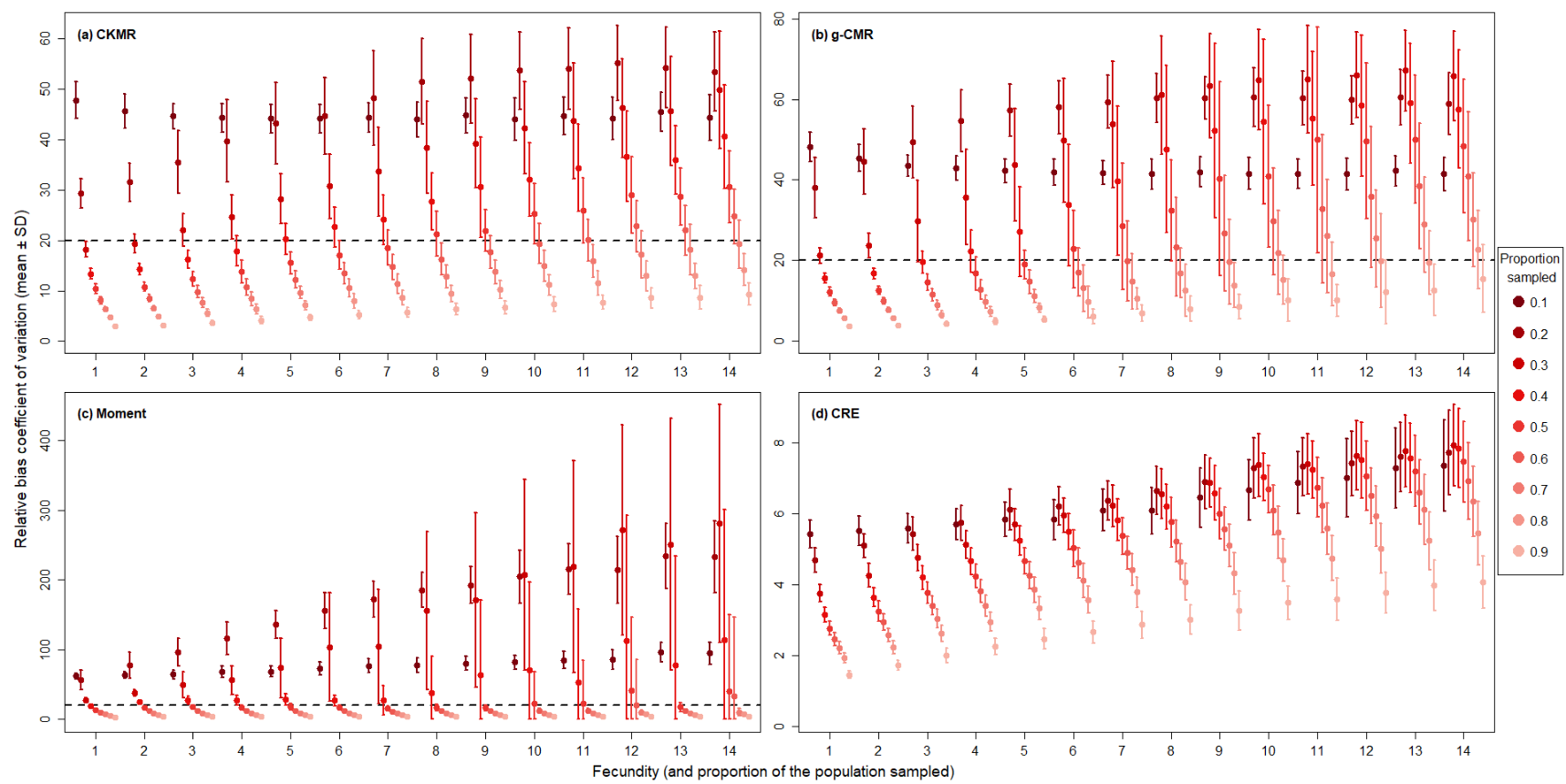


**Fig. S2** Relative bias (*N̂*/*N)* coefficient of variation, (mean ± SD) as a function of the fecundity (ranging from 1 to 12), and the proportion of the population sampled ranging from (0.1 to 0.9) for the (a) CKMR, (b) g-CMR, (c) Moment and (d) CRE methods. CKMR and CRE methods estimate adult population size while g-CMR and Moment methods estimate the whole population size and the breeding female population size, respectively. The long-dashed horizontal line represents the optimal value of 20% for a precise estimator, estimators above this line are too variable to be useful for wildlife management and conservation.


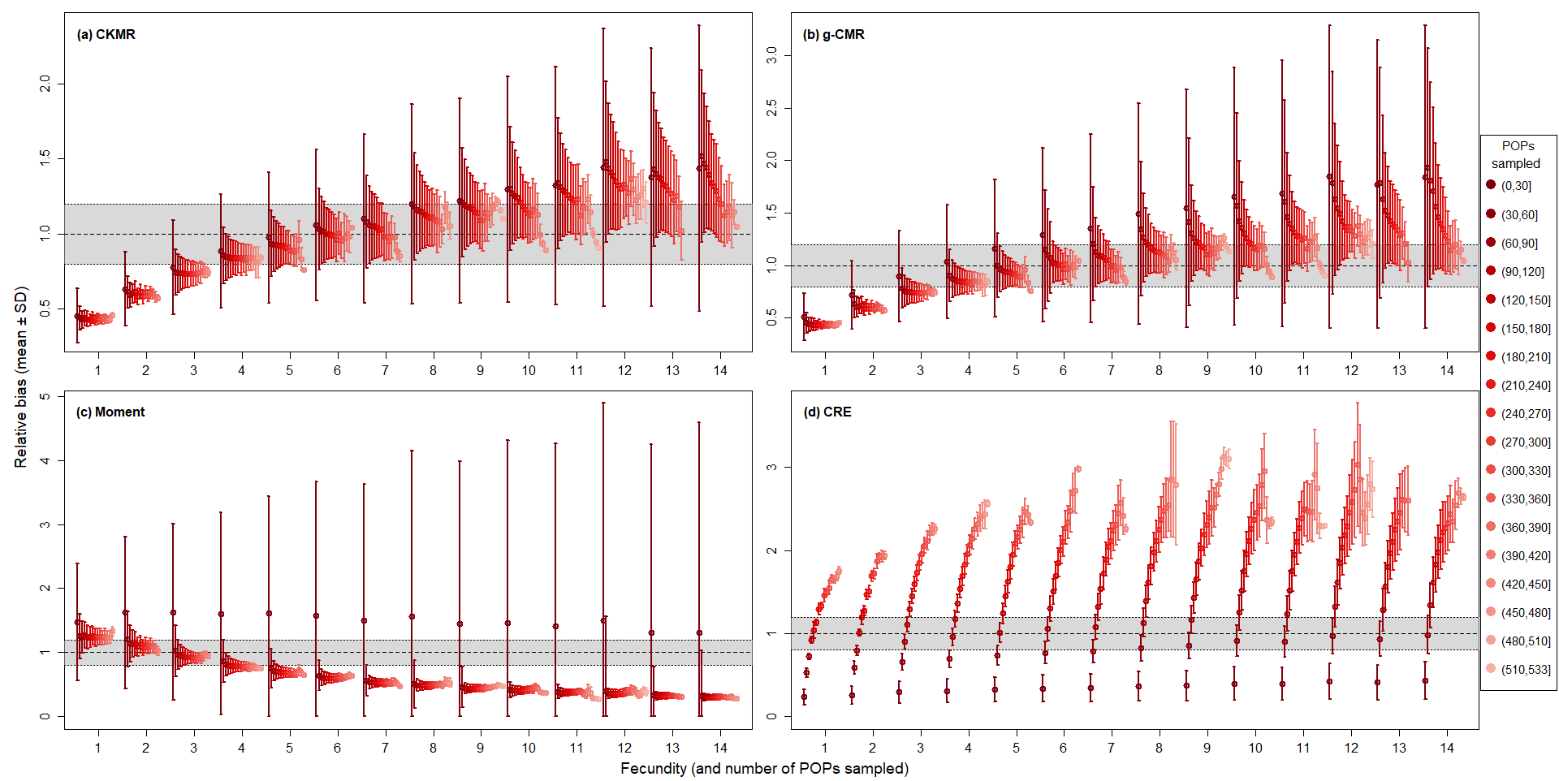


**Fig. S3** Relative bias (*N̂*/*N*, mean ± SD) as a function of the fecundity (ranging from 1 to 14), and the number of parent-offspring pairs (POP) sampled for the (a) CKMR, (b) g-CMR, (c) Moment and (d) CRE methods. CKMR and CRE methods estimate adult population size while g-CMR and Moment methods estimate the whole population size and the breeding female population size, respectively. The long-dashed horizontal line represents the optimal value for an unbiased estimator, estimators above this line are overestimating the true population size while estimators below are underestimating it. Points in the grey area represent estimations within 20% of the true population size.
